# Nonlinear scaling effects in the stiffness of soft cellular structures

**DOI:** 10.1098/rsos.181361

**Published:** 2019-01-16

**Authors:** Hayley Wyatt, Alexander Safar, Alastair Clarke, Sam L. Evans, L. Angela Mihai

**Affiliations:** 1 School of Engineering, Cardiff University, The Parade, Cardiff CF24 3AA, UK; 2 School of Mathematics, Cardiff University, Senghennydd Road, Cardiff CF24 4AG, UK

**Keywords:** cellular solids, neo-Hookean cell wall material, large-strain stretching, experimental testing, digital image correlation, finite element simulation

## Abstract

For cellular structures with uniform geometry, cell size and distribution, made from a neo-Hookean material, we demonstrate experimentally that large stretching causes nonlinear scaling effects governed by the microstructural architecture and the large strains at the cell level, which are not predicted by the linear elastic theory. For this purpose, three honeycomb-like structures with uniform square cells in stacked distribution were designed, where the number of cells varied, while the material volume and the ratio between the thickness and the length of the cell walls were fixed. These structures were manufactured from silicone rubber and tested under large uniaxial tension in a bespoke test fixture. Optical strain measurements were used to assess the deformation by capturing both the global displacements of the structure and the local deformations in the form of a strain map. The experimental results showed that, under sufficiently large strains, there was an increase in the stiffness of the structure when the same volume of material was arranged as many small cells compared to when it was organized as fewer larger cells. Finite element simulations confirmed our experimental findings. This study sheds light upon the nonlinear elastic responses of cellular structures in large-strain deformations, which cannot be captured within the linear elasticity framework.

## 1. Introduction

The design and assessment of cellular structures undergoing large elastic deformations is central in many industrial and biomedical applications, and their mathematical modelling and mechanical analysis pose many theoretical and computational challenges [1–5]. In particular, soft cellular structures are the subject of important research efforts in regenerative applications, such as soft tissue scaffolds, for which a better understanding of the mechanical behaviour is necessary to optimize their functional performance [6–13]. Cellular structures can also be found in both load-carrying and non-load-carrying matter, in nature and several industrial areas (e.g. impact protection, aerospace, microelectronics, pharmaceutical and food processes) [14–18]. Therefore, by studying the fundamental mechanical responses of cellular structures, important insights can be gained for the development of many areas of research.

For natural and man-made cellular structures, several key factors determine the magnitude of the enhancement of stress level in the cellular body, including the cell geometry, the cell wall thickness and the number of cells [1,2,4,5,19]. For two different structures made from the same volume of solid material, which is distributed uniformly as a small number of large cells or as a larger number of smaller cells, if the ratio between the thickness and the length of the cell walls is the same in both structures, then the stiffness of the structures under small strain elastic deformations is the same [15]. While this is valid for many cellular structures with linear elastic cell walls, and similarly, for structures with nonlinear elastic walls in the small strain regime, in many cellular solids, the cell size is expected to have a more independent effect on the elastic responses, even though this effect is typically obscured by other structural properties [20,21].

In this study, for cellular structures with uniform geometry, cell size and distribution, and a neo-Hookean hyperelastic cell-wall material [22,23], we demonstrate experimentally that sufficiently large stretching causes nonlinear elastic effects which are governed by the microstructural architecture and the large strains at the cell level, and are not predicted by the linear elasticity theory. For this purpose, three honeycomb-like structures with uniform square cells in stacked distribution were designed, where the number of cells varies, while the total material volume and the ratio between the thickness and the length of the cell walls are fixed. These structures were manufactured from silicone rubber and tested under large uniaxial tension in a bespoke test fixture. Optical strain measurement techniques were used to assess the deformation by capturing both the global displacements of the structure and the local deformations in the form of a strain map [24–28]. The experimental results showed that, under large strains, there was an increase in the stiffness of the structure when the same volume of material was arranged as many small cells compared to when it was organized as fewer larger cells. This behaviour is also captured by our finite element simulations of cellular structures with similar geometries and cell-wall material properties.

This study sheds light upon the nonlinear elastic responses [3,29–34] of soft cellular structures, which cannot be captured by the classical linear elastic theory. In particular, we show that, under sufficiently large strains, the stiffness of the structures with nonlinear elastic cell walls varies with the cell size [2,4], in contrast to the results predicted for structures with linear elastic cell walls [15], given that the same volume of material is used for each structure, and that the thickness-to-length ratio for the cell walls remains the same. This has important implications for the optimal design of cellular materials in various applications, and in particular, for stretch-dominated architectures, which are structurally more efficient, due to a higher stiffness-to-weight ratio, than the bending-dominated ones [3–5,15,19,35].

## 2. Experimental material and methods

### 2.1. Structure design and manufacture

**Figure 1.**
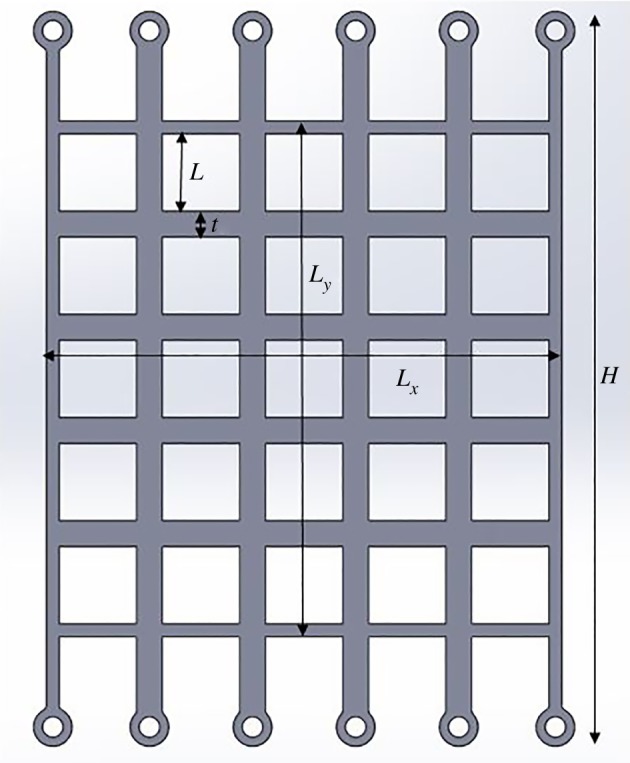
Geometry of cellular structure with 5 × 5 cells.

Three periodic honeycomb-like structures with a different number of square cells in stacked distribution were designed and manufactured, ensuring that the overall volume of solid material used and the ratio between the thickness and the length of the cell walls are the same for all structures, while the number of cells varies. The geometric parameters for the designed structures are summarized in table 1 and illustrated on a single structure in figure 1. In this figure, the tabs seen at the top and the bottom of the structure allow for the physical structure to be mounted in the bespoke test fixture, as described in detail in the next section which focuses on the experimental set-up.

**Table 1.** Geometric parameters of the undeformed cellular structures tested experimentally.

cellular structure	overall height, *H* (mm)	structure width, *L_x_* (mm)	structure height, *L_y_* (mm)	structure depth, *L_z_* (mm)	cell-wall length, *L* (mm)	cell-wall thickness, *t* (mm)	cell-wall thickness-to-length ratio, *t*/*L*
3 × 3 cells	170.833	100	100	10	25.000	8.333	3.000
5 × 5 cells	142.500	100	100	10	15.000	5.000	3.000
9 × 9 cells	127.667	100	100	10	8.333	2.778	3.000

Individual aluminium moulds were created for each of the structures, and the structures were then cast out of Tech-Sil 25 silicone. This silicone is a two-part silicone, mixed as per the manufacturer's instructions, which underwent a two-part degassing process, first after the initial mixing and second after the casting, then allowed 24 h to cure. The material behaviour of this silicone is characterized by a neo-Hookean hyperelastic model, described by equation (4.1), with Young's modulus *E* = 0.74 MPa and Poisson's ratio *ν* = 0.48 under infinitesimal deformations. The neo-Hookean model is the simplest nonlinear hyperelastic model, originally proposed to characterize the nonlinear elastic behaviour of rubberlike material in [22,23]. Our parameter values were obtained through uniaxial tensile and compression testing, using the process of inverse analysis, prior to the manufacturing of the structures. A total of six structures were manufactured, two of each structure type.

### 2.2. Experimental set-up

**Figure 2.**
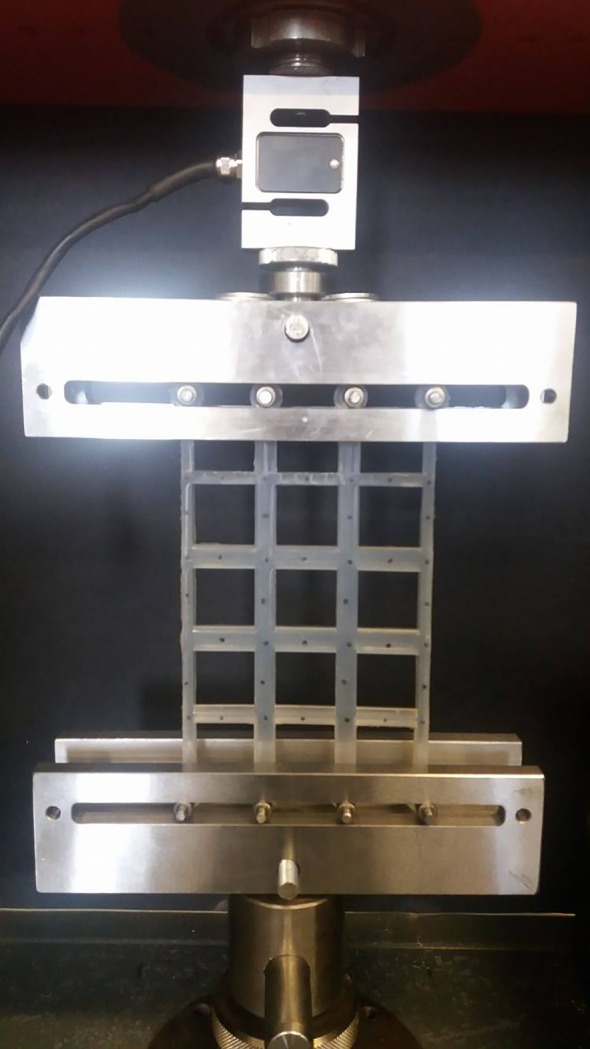
Bespoke test fixture allowing the structure to slide in the horizontal direction and create a straight edge during tension.

To conduct the uniaxial tensile testing of each structure, a bespoke fixture was designed (figure 2). This allowed for the (top and bottom) ends of the structure to slide horizontally, while the structure was loaded vertically, meaning that all the initially straight and vertical cell walls remained almost straight and vertical throughout the testing, avoiding the unwanted bending of the side walls, which is commonly seen during more traditional tensile tests whereby the ends of a structure are clamped. This was achieved through the use of dowel rods and needle roller bearings, where the friction within the system was minimized through polishing of the contact surfaces. When the coefficient of friction was experimentally measured for the system, a resulting mean CoF of 0.02 ± 0.003 was found, demonstrating minimal friction within the bespoke test fixture.

Uniaxial tensile tests were conducted using a Zwick–Roell Z050 tensile testing machine, with a 2 kN load cell to measure tensile force. Initially, loading and unloading tests were carried out to verify that the structures were elastically deformed, i.e. all the changes in the deformed structures were reversible. For this, each structure was subjected to a 60 N tensile load in 10 N increments and unloaded to 0 N after each increment. To measure their local and global deformations, the structures were subjected to a maximum tensile load of 50 N. To capture quasi-static deformations, the tests were performed at a velocity of 2 mm s^−1^, and a pre-load of 1 N was used to remove slack from the experimental set-up. Tests were conducted twice for each structure type (*n* = 2).

### 2.3. Digital image correlation

Digital image correlation (DIC) is a non-contact optical measurement technique measuring specimen displacement. A high-contrast pattern is applied to the surface of the specimen, which provides unique points of identification to allow the software to track the displacement of the specimen. The specimen is imaged in its unloaded state, and this acts as a point of reference for the software. The specimen is then imaged throughout loading, either through video or through a series of camera images. The software will then use the captured images to track the unique points within the high-contrast pattern, measuring the displacement of the specimen. From the displacement, strain can then be computed using the parameter of the affine transformation and the gradients of the deformation [24–28]. Within this study, two DIC systems were used, the first was the Imetrum Video Gauge system. This was used to capture the global deformation of the structure through the application of virtual strain gauges. The second system was a Q-400 Dantec Dynamic system used to create a two-dimensional map of the strains at a local level, focusing solely on the centre cell of each structure.

#### 2.3.1. Measuring global deformation

A video strain gauge system (Imetrum) was used to capture the global deformation of the structure during tensile tests. The system was used with a single camera with a general-purpose lens and calibrated using markers of a known distance apart within the field of view, as per the manufacturer's instructions. Markers were applied to the surface of the specimen to allow the software, provided as part of the system, to track the deformation of the structure. These were applied to the structure using a black marker pen, with markers applied to the intersections and the mid-wall of the cells. When processing the data, virtual strain gauges were applied to the structure, using markers that maximized the length of the gauge, thus reducing errors within the system. For the purpose of this study, a limited number of points were selected to validate the new loading fixture, although there is a potential for further exploiting results obtained from these data.

#### 2.3.2. Measuring local deformation

The Q-400 (Dantec Dynamics) system was used to capture the local deformation of the structures and to validate the finite element models (FEMs). The system consisted of the necessary software, Instra4D, a HiLis light source and a data logging system to connect the cameras to the laptop. The HiLis light source is a high-intensity LED illumination system which provides cool and homogeneous illumination. Two digital cameras were used with the system and were mounted onto a tripod with the HiLis light source positioned between them. The two cameras were connected to a data logging system, which, in turn, was connected to the laptop. Following this, the aperture and focus of both cameras were adjusted, focusing on the high-contrast speckle pattern applied to the surface of the silicone specimen [27]. This high-contrast speckle pattern was applied using white and black face paint (Snazaroo). Three different camera set-ups were required, due to the differences in structure geometry, ensuring the most appropriate set-up for each structure in terms of the field of view. Each camera set-up differed in terms of their field of view only, with the same equipment including calibration target and camera lenses used for each. Following the set-up of the DIC system, it was necessary to conduct a calibration. This determined the position and orientation of each of the cameras with respect to the surface of the specimen and related the pixel size of the object image to the metric scale. To calibrate the system, a series of eight calibration images were taken of a calibration target. The calibration target used for this study was a 9 × 9 grid, 40 × 40 mm (Dantec Dynamics). The target was rotated and tilted for each image to allow the software to determine the required parameters. A calibration residuum of less than 0.1 was considered acceptable [27]. Data were processed within the DIC software, Instra4D, with the parameters from data capture and processing displayed in table 2. For each structure, two polygons were drawn over the surface of the struts, one covering the vertical strut and one covering the horizontal strut. In both cases, the joints were excluded from the analysis. For the polygon, mean values of the strain over the surface were exported. Throughout this study, the Green–Lagrange strain is used [3,30,33].

**Table 2.** Parameters for the DIC data capture and processing.

experimental technique used	3 × 3 cell structure	5 × 5 cell structure	9 × 9 cell structure
calibration residuum	<0.1	<0.1	<0.1
speckle pattern size	0.45–1.2 mm	0.35–1.2 mm	0.25–0.6 mm
subset	17 pixels	17 pixels	17 pixels
step size	17 pixels	17 pixels	17 pixels
spatial resolution	1.33 mm	1.02 mm	0.697 mm
total number of images	15	15	15
displacement			
displacement noise	0.005 mm	0.015 mm	0.020 mm
strain			
smoothing method	none	none	none
strain noise	20 mstrain	20 mstrain	20 mstrain

## 3. Experimental results

### 3.1. Global deformation of structures

**Figure 3.**
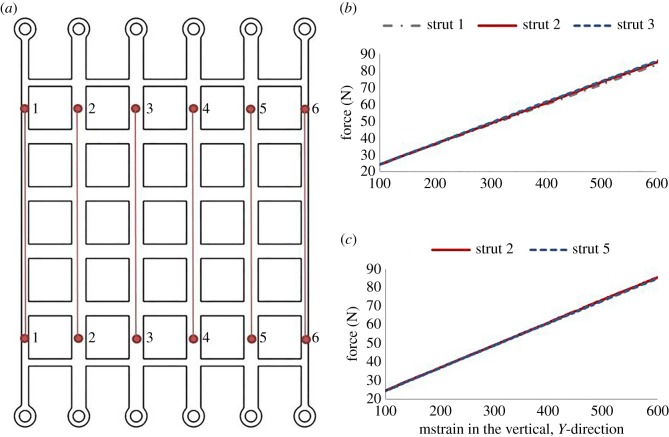
Comparison of vertical strains in vertical struts: (*a*) schematic of cellular structure showing the location and numbering of strain gauges for each strut; (*b*) comparison of mean vertical strains in struts 1, 2 and 3 and (*c*) comparison of mean vertical strains in struts 2 and 5. The strain shown is in mstrain.

To validate the new loading fixture (figure 2), and thus ensure that the boundary conditions achieved are as expected, the global behaviour of each structure was analysed using the Imetrum video strain gauge system. Virtual strain gauges were added to the structure, as shown in figure 3*a*, with the red dots depicting the marks made on the physical structure. Struts 1, 2 and 3 were compared to verify that the strain was the same for each of these struts, thus ensuring that no end-effects were present in the structure. The results can be seen in figure 3*b*, where the struts exhibit almost identical mean vertical strains at loads up to around 80 N. Throughout the paper, strains are presented as millistrain or mstrain. Struts 2 and 5 were analysed to ensure that the new loading fixture created symmetrical boundary conditions, as seen in figure 3*c*, where the mean vertical strains for these two struts are almost identical.

**Figure 4.**
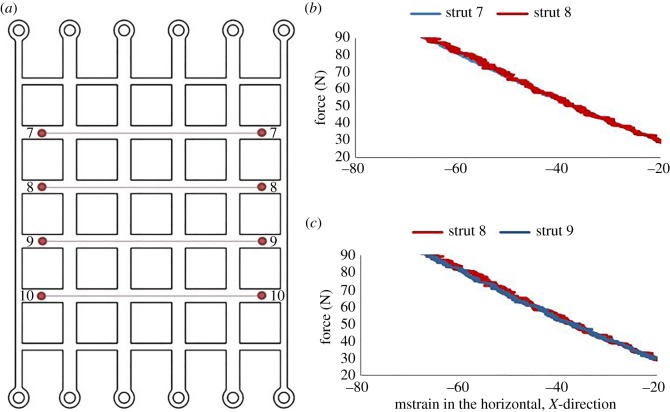
Comparison of horizontal strains in horizontal struts: (*a*) schematic of cellular structure showing the location and numbering of strain gauges for each horizontal strut; (*b*) comparison of mean horizontal strains in struts 7 and 8 and (*c*) comparison of mean horizontal strains in struts 8 and 9. The strain shown is in mstrain.

In addition to the vertical strain of different struts, the horizontal strain was also analysed. Virtual strain gauges were added to the structure as shown in figure 4*a*, with the red dots depicting the marks made on the physical structure. Struts 7 and 8 were compared to verify that the strain was the same for each strut, thus ensuring that no end-effects were present within the deformed structure. These results are illustrated in figure 4*b*, where the struts exhibit almost identical mean horizontal strains at loads up to around 80 N. Struts 8 and 9 were analysed to ensure that the loading fixture created symmetrical boundary conditions, as seen in figure 4*c*, where the mean horizontal strains for these two struts are almost identical.

Although the reported results correspond to the structure with 5 × 5 cells, similar results were obtained for the structures with 3 × 3 and 9 × 9 cells, thus validating the new loading fixture in creating the desired boundary conditions for uniaxial tensile testing under large strains.

**Figure 5.**
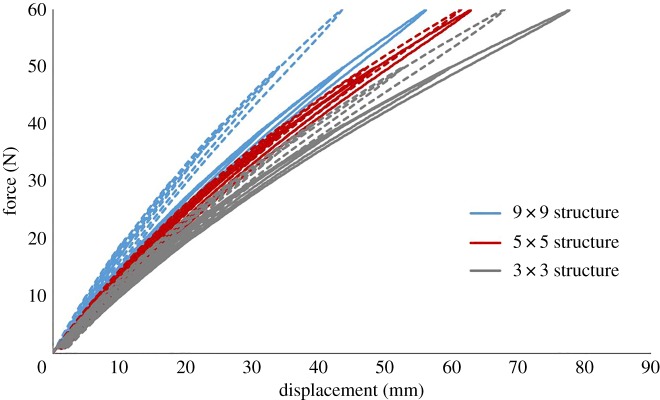
Applied force versus maximum vertical displacement in tensile loading and unloading of the three structures. The blue, red and grey lines represent the 9 × 9 structure, the 5 × 5 structure and the 3 × 3 structure, respectively, with the dotted and solid lines differentiating between different samples. For experimental testing, *n* = 2.

To ensure that all testing remained within the elastic limits of the structures and no plastic deformation occurred, a series of loading–unloading tests were also performed. The loading conditions were as described previously, with each structure being subjected to a 60 N tensile load in 10 N increments and unloaded to 0 N after each increment. Figure 5 shows the results for the three structures. The data demonstrate no plastic deformation of the structure, with each structure clearly remaining within its elastic region, with minimal hysteresis between the loading and unloading paths.

Additionally, the experimental results demonstrated some variation between different samples of the same structure (figure 5). Despite these variations, the same trend was seen within the data, with the 9 × 9 structure being stiffer than the 5 × 5 structure which was, in turn, stiffer than the 3 × 3 structure. The variation between samples could be caused by a number of factors. One possibility is the slight inconsistencies in the manufacturing process. For example, the silicone used is a two-part silicone and small variations in the volume of the mixture could influence its mechanical properties. Another possible explanation for the variation in experimental results is the slight change in the testing environment, for example, tests were not conducted in a temperature-controlled environment.

### 3.2. Local deformation of structures

**Figure 6.**
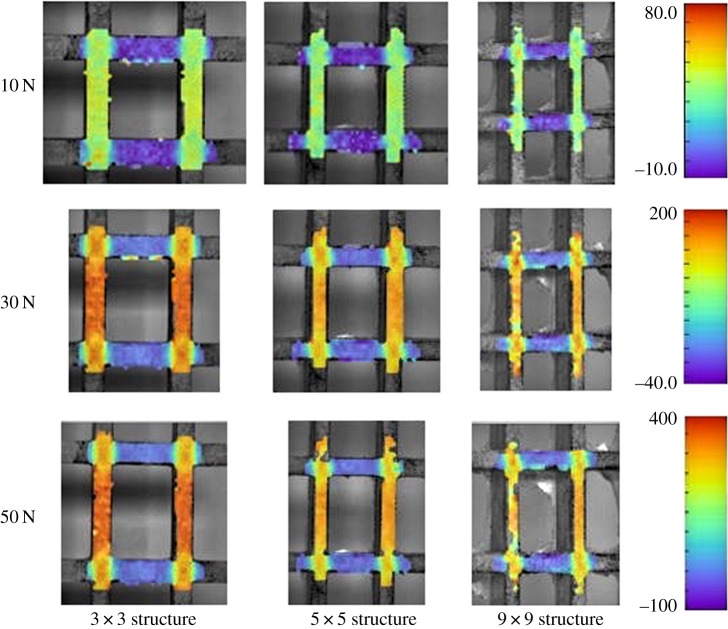
Colour maps showing the vertical strain in each structure at 10, 30 and 50 N, with the left-hand column depicting the 3 × 3 structure, the middle column depicting the 5 × 5 structure and the right-hand column depicting the 9 × 9 structure. The strain shown is in mstrain.

**Figure 7.**
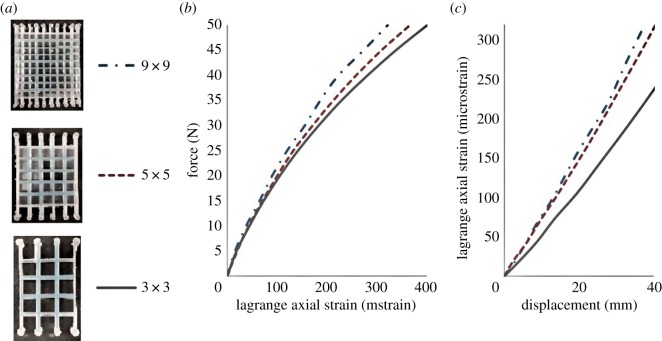
Comparison of the deformation of the centre cell of the structure tested experimentally using DIC: (*a*) photos of physical structures; (*b*) applied tensile force versus mean vertical strain; (*c*) mean vertical strain versus maximum vertical displacement of the load machine. The strain shown is in mstrain.

**Figure 8.**
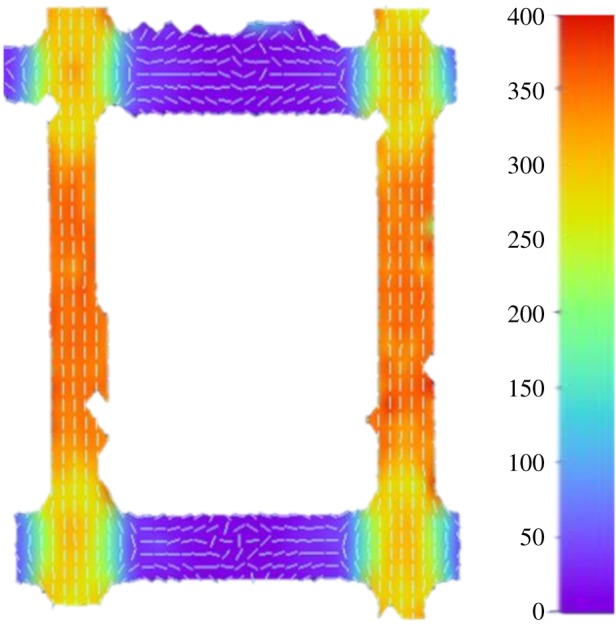
The maximum principal strain for the structure with 3 × 3 cells at a 50 N tensile load, with the small lines within the strain map showing the local orientation of the strain. The strain shown is in mstrain.

As all cells in the structure are deformed similarly, for our analysis, we focus on the central cell. In figures 6–8, we show the strain maps within the three different structures under three different loads each. The same strain map scale was used for the three structures within each image, but varied for the different loading stages. At 10 N tensile load, there appears to be almost no difference between the strains in the three structures, as seen from figure 6, but as the load increases, the difference between the strains in these structures increases. For example, figure 6 suggests that, in the structure with 9 × 9 cells, the vertical strain at 50 N tensile load is greater than that in the 5 × 5 cell structure, which, in turn, is greater than in the 3 × 3 cell structure.

This observation is confirmed by the results plotted in figure 7, where the force required to stretch the structure with 9 × 9 cells to a certain magnitude of Lagrange axial strain (or by a certain maximum vertical displacement) is greater than for the structure with 5 × 5 cells, which, in turn, is greater than for the structure with 3 × 3 cells. Importantly, it should be noted that figure 7*b* shows almost no differences in the strains of the cell walls within the small strain regime (typically, this is classified as below 4% strain or 40 mstrain). The strain within the cell walls begins to vary at around 150 mstrain, showing the stiffening effect of the cell arrangements at larger deformations.

To illustrate the orientation of the maximum principal strains in the structures under tensile loads, in figure 8, the strain orientation in the structure with 3 × 3 cells at a 50 N tensile load is shown. However, similar trends were also observed in the other structures, although with different magnitudes of strain. As seen from figure 8, the maximum principal strain is orientated in the vertical direction within the vertical struts and corresponds to longitudinal tension, whereas in the horizontal walls, the maximum principal strain is orientated in the horizontal direction and corresponds to longitudinal compression. At the intersection between the horizontal and vertical walls, the orientation of the maximum principal strain shows a more curved alignment, highlighting the more complex behaviour that occurs at these joints.

## 4. Finite element simulation

### 4.1. Model set-up

In this section, we assess computationally nonlinear stretching effects in periodic cellular structures with square cells in stacked distribution and neo-Hookean cell wall material [22,23], similar to those tested experimentally. Within the finite element simulations, the generalized neo-Hookean model was used, characterized by the strain energy density function:

4.1 $$\omega (I_{1},I_{2},I_{3})=\;\frac{\mu }{2}(I_{1}-3-\ln\;I_{3})+\frac{\lambda }{2}{\left(\ln\;I_{3}^{1/2}\right)}^{2}$$

In equation (4.1), *μ* = *E*/[2(1 + *ν*)] > 0 and *λ* = *νE*/[(1 + *ν*)(1–2*ν*)] > 0 are constant material parameters, with *E* and *ν* denoting the Young's modulus and Poisson's ratio, respectively, and *μ* representing the shear modulus at infinitesimal deformations, and *I*_1_,*I*_2_,*I*_3_ are the principal invariants of the Cauchy–Green deformation tensors **C** = **F**^T^**F** and **B** = **FF**^T^, with **F** denoting the (large-strain) deformation gradient. The Green–Lagrange strain tensor then takes the form **E** = (**C** − **I**)/2, where **C** is the right Cauchy–Green deformation tensor defined above and **I** is the identity tensor (note the boldface notation for tensors) [3,30,33].

As in the experimental tests, a Young's modulus, *E*, of 0.74 MPa and a Poisson's ratio, *ν*, of 0.48 were assumed for the cell-wall material. These parameter values were then used to compute the constants *μ* and *λ* for the neo-Hookean model, given by (4.1), in the finite element simulations. As a Poisson's ratio of 0.5 corresponds to perfect incompressibility, a Poisson's ratio of 0.48 represents a condition of slight compressibility (or near incompressibility).

**Figure 9.**
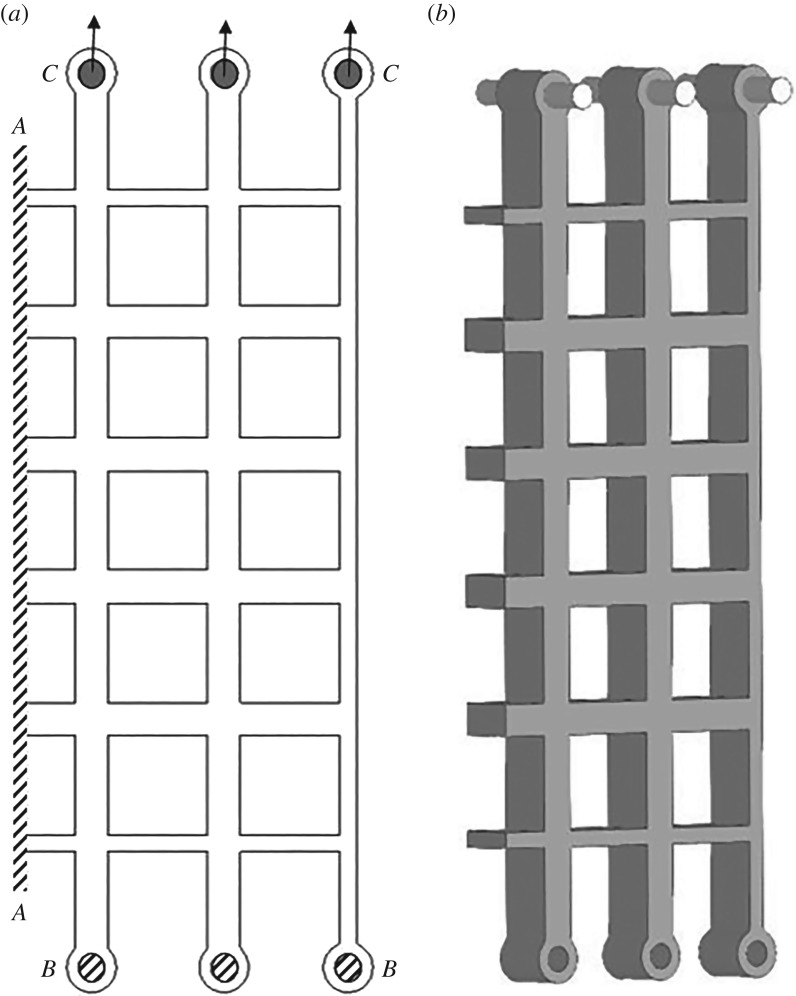
(*a*) Schematic view of the finite element boundary conditions, with dash lines along *AA* showing the surfaces fixed using the symmetry constraint, *BB* showing the fixed constraint applied in the horizontal and out of plane direction for the inner surface of the holes and *CC* showing the location of the rigid rods and the direction of stretch. (*b*) A three-dimensional view of the finite element set-up, clearly displaying the location of the rigid rods.

The modelled structures had the same geometry as those tested experimentally, but the symmetry of the boundary conditions was used to reduce computational cost, modelling only half of the tested specimen. To compute the force–displacement responses in the computational structures, cylindrical metal rods inserted through the hoops at the end of the structure were modelled, mimicking the physical tests conducted experimentally. The position of these rods can be seen clearly in figure 9. These rods were modelled as rigid bodies, and the boundary conditions between the rods and the structure, as rigid interfaces. The rigid rods had prescribed displacement in the positive vertical direction to create the prescribed vertical stretch of the structure. The internal and external faces of the structure were allowed to deform freely. The boundary conditions applied to the model are shown schematically in figure 9.

**Figure 10.**
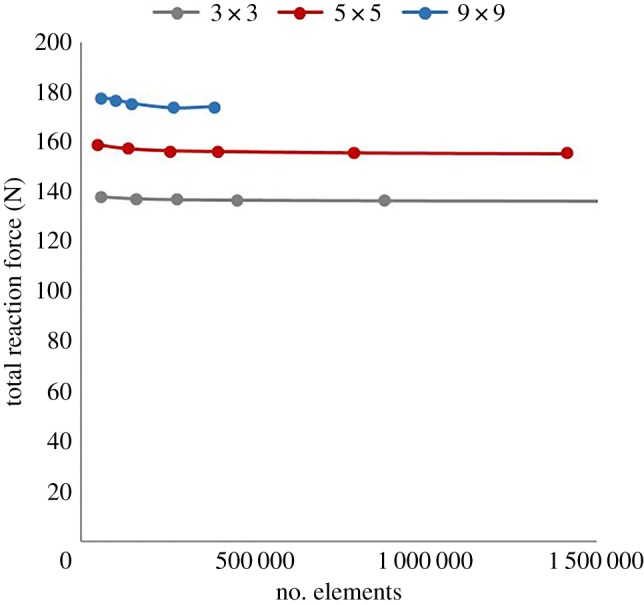
Mesh sensitivity study for the finite element modelling of the three structures investigated, showing the total reaction force for each structure and the total number of elements in each model.

The numerical results recorded here were obtained within the finite element for biomechanics (FEBio) software environment [36]. The model structures were created in SolidWorks and imported into the FEBio software, and a mesh refinement study was performed for each structure, the results of which are shown in figure 10, to ensure that the numerical results are independent of the mesh size. To evaluate the mesh sensitivity, the total reaction force was used as this was a criterion of interest in evaluating the overall behaviour of each structure. The reaction force was computed within the FEBio software for each of the rigid rods used in constraining the structures. To calculate the total reaction force for each structure, the computed forces for each rigid rod were added together and the resulting reaction force was doubled due to the symmetry assumption applied to the model.

**Figure 11.**
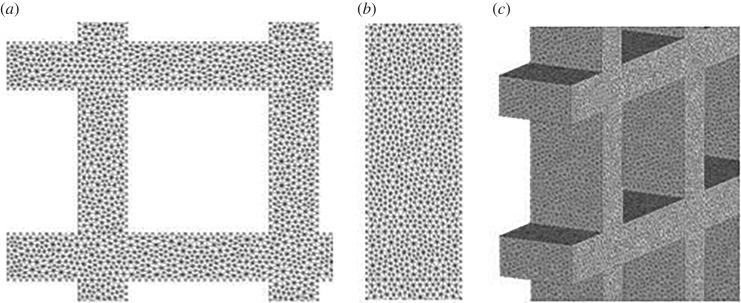
Example of the mesh for the 5 × 5 structure with (*a*) showing the front view of the mesh, (*b*) showing the side-on view or the elements through the thickness and (*c*) showing a three-dimensional view of the meshed structure.

The results were deemed to be independent of meshing parameters once three results in a row were within 1% of one another. The mesh elements used were four-node linear tetrahedral elements, with the exact details of the mesh used for each structure found in table 3. An example of the mesh used for the 5 × 5 structure can be seen in figure 11.

**Table 3.** Final mesh parameters for each structure.

structure	total number of elements	total number of nodes	element type
3 × 3	449 851	98 921	four-node linear tetrahedral
5 × 5	398 165	94 147	four-node linear tetrahedral
9 × 9	267 391	72 971	four-node linear tetrahedral

### 4.2. Comparison with experimental data

**Figure 12.**
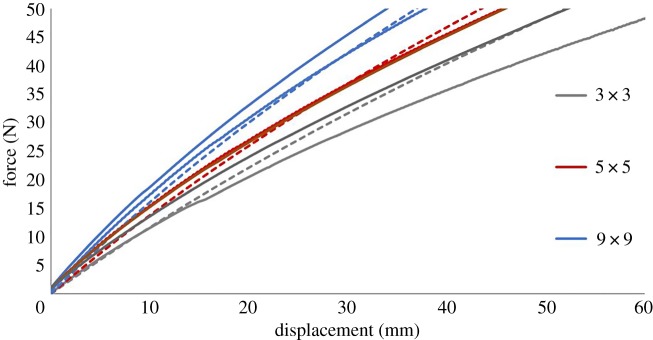
Comparison of force–displacement curves for the FEBio computational models (dashed lines) and the corresponding experimental data (solid lines) of the three structures. For experimental testing, *n* = 2.

**Figure 13.**
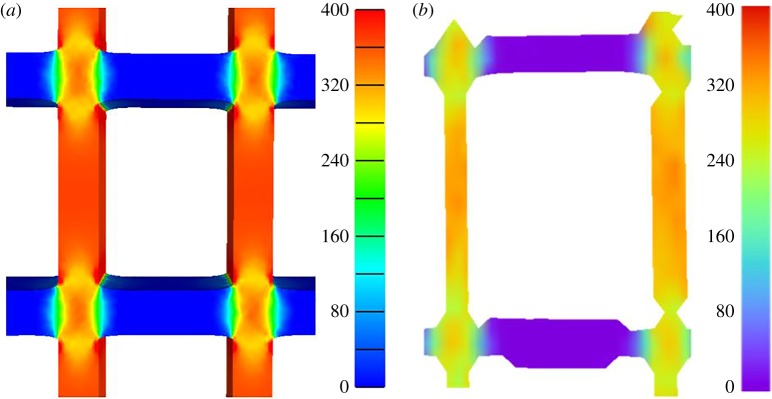
Comparison between vertical strain in the structure with 5 × 5 cells at 50 N load: (*a*) FEBio model and (*b*) experimental. Note that the two figures are shown at similar scale bars (the colours are software-specific). The strain shown is in mstrain.

The numerical results from the computational models were compared with the experimental data. For the finite element models, the reaction force on the rigid rods was exported, as well as the displacement of the structure, to compare with the force–displacement data acquired experimentally. The results shown in figure 12 for the finite element simulation are in qualitative agreement with the experimental data; as for both the computation and experimental structures, the stiffness clearly increases with the number of cells. In addition to the force–displacement curves, the vertical strain maps across the computational and experimental structures with 5 × 5 cells are presented, at the same scale, in figure 13. In this figure, the magnitudes of the strains found computationally and experimentally are similar.

For all structures, there are slight differences between the finite element simulations and experimental results. These differences can be attributed to assumptions made within the modelling process. One example would be the material model representing the silicone used to create the structures. For this study, a neo-Hookean hyperelastic model was chosen with parameters determined experimentally. Furthermore, there is a variation in the experimental results between samples making it difficult to evaluate the difference between finite element simulations and experimental testing. More experimental testing is required to assess these variations.

### 4.3. Analysis of finite element models

**Figure 14.**
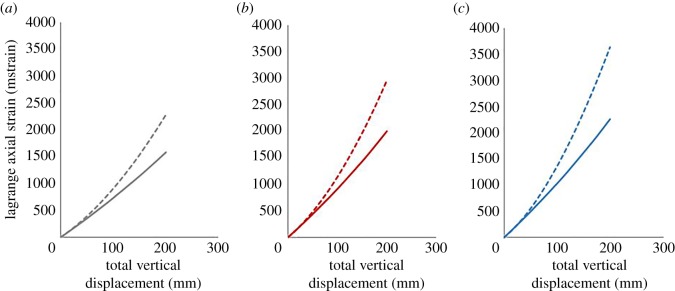
Additional analysis of the finite element models for each structure, with (*a*) showing the 3 × 3 structure, (*b*) showing the 5 × 5 structure and (*c*) showing the 9 × 9 structure. The solid lines show the response of the centre cell joint and the dashed lines show the response of the centre cell vertical wall. Each figure shows the total structure displacement against the Lagrange axial strain (mstrain).

To understand the difference in behaviour between the different structures investigated as part of this study, further analysis of the finite element models was conducted. As part of this analysis, it was found that the joints within the structures exhibit a different nonlinear behaviour compared to the cell walls (figure 14). The nonlinear behaviour of the joints changes due to the extra constraints; therefore, when the number and size of the joints change within a structure, the mechanical response of the structure changes. Figure 14 shows that, in the small strain regime, the difference between the response of the cell joint and the cell wall is negligible, with the difference increasing outside of the small strain regime. This trend can be seen in all structures investigated as part of this study.

## 5. Conclusion

In general, for different cellular structures with linear elastic cell walls, containing the same volume of solid material, which is distributed either as a small number of large cells or as a larger number of smaller cells, when the ratio between the thickness and the length of a wall is the same, the stiffness of the corresponding structure is expected to be the same [15]. For similar structures of nonlinear elastic material also, this behaviour appears reasonable under small strains. However, in real structures, under sufficiently large strains, the cell size is expected to have a more independent effect, even though this effect may be relatively minor or harder to separate from other mechanical responses [20,21].

The aim of this paper was to separate the cell size effect from other nonlinear elastic responses when the size of the cells and the size of the structure are comparable (i.e. the cells are not infinitesimally small relative to the cellular sample). Specifically, for cellular structures with uniform cell size, shape and distribution, we demonstrated experimentally, for the first time, that, under large-strain deformations, the stiffness in cell walls made from an isotropic nonlinear hyperplastic material increases when the number of cells increases, while the volume of solid material and the ratio between the thickness and the length of the wall remain fixed. This can be attributed to the nonlinear responses of the elastic cell-wall joints in addition to that of the cell walls. Therefore, when the number and size of the joints change, the response of the structure will change. Further investigation is required to understand the limits of the structures in regard to their stiffness.

For our experimental tests, we developed a novel loading method for cellular structures under uniaxial tensile tests, which allows for a structure to be loaded in such a way that the end-effects are minimal and the boundary conditions are suitable for nonlinear elastic analysis under large strains.

In addition to the experimental study, we constructed computational models which simulate the physical structures and reproduce the elastic effects observed experimentally. FEMs are suitable for further investigation of three-dimensional (3D) structures with different cell size or cell wall material parameters, and subject to different loads [4,5].

Although many natural structures are irregular, cellular structures with regular geometry are easily reproducible and can be studied systematically to identify the independent influence of different properties [1,2,4,5,19]. In particular, our analysis offers valuable insights into the independent mechanical effect of cell size for structures under large elastic strains, which cannot be captured within the linear elasticity framework. Our results naturally open the door to many new questions and will inspire further theoretical and experimental investigations.

## Supplementary Material

Supplementary Material

## Supplementary Material

Supplementary Material 2

## Data accessibility

The datasets supporting this article have been uploaded as part of the electronic supplementary material.
